# ACE I/D genotype associates with strength in sarcopenic men but not with response to ACE inhibitor therapy in older adults with sarcopenia: Results from the LACE trial

**DOI:** 10.1371/journal.pone.0292402

**Published:** 2023-10-20

**Authors:** Christos Rossios, Tufail Bashir, Marcus Achison, Simon Adamson, Asangaedem Akpan, Terry Aspray, Alison Avenell, Margaret M. Band, Louise A. Burton, Vera Cvoro, Peter T. Donnan, Gordon W. Duncan, Jacob George, Adam L. Gordon, Celia L. Gregson, Adrian Hapca, Cheryl Hume, Thomas A. Jackson, Simon Kerr, Alixe Kilgour, Tahir Masud, Andrew McKenzie, Emma McKenzie, Harnish Patel, Kristina Pilvinyte, Helen C. Roberts, Avan A. Sayer, Karen T. Smith, Roy L. Soiza, Claire J. Steves, Allan D. Struthers, Divya Tiwari, Julie Whitney, Miles D. Witham, Paul R. Kemp

**Affiliations:** 1 Cardiovascular and Respiratory Interface Section, National Heart and Lung Institute, Imperial College London, South Kensington Campus, London, United Kingdom; 2 Tayside Clinical Trials Unit (TCTU), Tayside Medical Science Centre (TASC), University of Dundee, Ninewells Hospital & Medical School, Dundee, United Kingdom; 3 University of Liverpool, Liverpool University Hospitals NHS FT Trust, Clinical Research Network Northwest Coast, Liverpool, United Kingdom; 4 AGE Research Group, NIHR Newcastle Biomedical Research Centre, Translational Clinical Research Institute, Newcastle University, Cumbria Northumberland Tyne and Wear NHS Foundation Trust and Newcastle upon Tyne Hospitals NHS Trust, Newcastle upon Tyne, United Kingdom; 5 Health Services Research Unit, University of Aberdeen, Aberdeen, United Kingdom; 6 Medicine for the Elderly, NHS Tayside, Dundee, United Kingdom; 7 Ageing and Health, University of Dundee, Dundee, United Kingdom; 8 Victoria Hospital, Kirkcaldy, United Kingdom; 9 Centre for Clinical Brain Sciences, University of Edinburgh, Edinburgh, United Kingdom; 10 Division of Population Health and Genomics, School of Medicine, University of Dundee, Dundee, United Kingdom; 11 Medicine for the Elderly, NHS Lothian, Edinburgh, United Kingdom; 12 Dept Clinical Pharmacology, Division of Molecular & Clinical Medicine, University of Dundee Medical School, Ninewells Hospital, Dundee, United Kingdom; 13 Unit of Injury, Inflammation and Recovery, School of Medicine, University of Nottingham, Nottingham, United Kingdom; 14 NIHR Nottingham Biomedical Research Centre, Department of Medicine for the Elderly, University Hospitals of Derby and Burton NHS Foundation Trust, Derby, United Kingdom; 15 Musculoskeletal Research Unit, Bristol Medical School, University of Bristol, Bristol, United Kingdom; 16 Older Person’s Unit, Royal United Hospital NHS Foundation Trust Bath, Bath, United Kingdom; 17 Institute of Inflammation and Ageing, University of Birmingham, Birmingham, United Kingdom; 18 Department of Older People’s Medicine, Newcastle upon Tyne Hospitals NHS Foundation Trust, Newcastle upon Tyne, United Kingdom; 19 Ageing and Health Research Group, Usher Institute, University of Edinburgh, Edinburgh, United Kingdom; 20 Clinical Gerontology Research Unit, Nottingham University Hospitals NHS Trust, City Hospital Campus, Nottingham, United Kingdom; 21 NIHR Biomedical Research Centre, University of Southampton and University Hospital Southampton NHSFT, Southampton, Hampshire, United Kingdom; 22 Academic Geriatric Medicine, University of Southampton, Mailpoint 807 Southampton General Hospital, Southampton, United Kingdom; 23 Ageing & Clinical Experimental Research (ACER) Group, University of Aberdeen, Aberdeen, United Kingdom; 24 Department of Twin Research and Genetic Epidemiology, King’s College London & Department of Clinical Gerontology, King’s College Hospital, London, United Kingdom; 25 Bournemouth University and Royal Bournemouth Hospital, Bournemouth, United Kingdom; 26 School of Population Health & Environmental Sciences, King’s College London and King’s College Hospital, London, United Kingdom; Fujita Health University, JAPAN

## Abstract

**Background:**

Angiotensin II (AII), has been suggested to promote muscle loss. Reducing AII synthesis, by inhibiting angiotensin converting enzyme (ACE) activity has been proposed as a method to inhibit muscle loss. The LACE clinical trial was designed to determine whether ACE inhibition would reduce further muscle loss in individuals with sarcopenia but suffered from low recruitment and returned a negative result. Polymorphic variation in the ACE promoter (I/D alleles) has been associated with differences in ACE activity and muscle physiology in a range of clinical conditions. This aim of this analysis was to determine whether I/D polymorphic variation is associated with muscle mass, strength, in sarcopenia or contributed to the lack of response to treatment in the LACE study.

**Methods:**

Sarcopenic individuals were recruited into a 2x2 factorial multicentre double-blind study of the effects of perindopril and/or leucine versus placebo on physical performance and muscle mass. DNA extracted from blood samples (n = 130 72 women and 58 men) was genotyped by PCR for the ACE I/D polymorphism. Genotypes were then compared with body composition measured by DXA, hand grip and quadriceps strength before and after 12 months’ treatment with leucine and/or perindopril in a cross-sectional analysis of the influence of genotype on these variables.

**Results:**

Allele frequencies for the normal UK population were extracted from 13 previous studies (I = 0.473, D = 0.527). In the LACE cohort the D allele was over-represented (I = 0.412, D = 0.588, p = 0.046). This over-representation was present in men (I = 0.353, D = 0.647, p = 0.010) but not women (I = 0.458, D = 0.532, p = 0.708). In men but not women, individuals with the I allele had greater leg strength (II/ID = 18.00 kg (14.50, 21.60) vs DD = 13.20 kg (10.50, 15.90), p = 0.028). Over the 12 months individuals with the DD genotype increased in quadriceps strength but those with the II or ID genotype did not. Perindopril did not increase muscle strength or mass in any polymorphism group relative to placebo.

**Conclusion:**

Our results suggest that although ACE genotype was not associated with response to ACE inhibitor therapy in the LACE trial population, sarcopenic men with the ACE DD genotype may be weaker than those with the ACE I/D or II genotype.

## Introduction

The normal human growth cycle includes periods of muscle growth, muscle maintenance and muscle loss [1]. As people live to a greater age through improved environmental conditions and medical therapies, the period of loss is prolonged. Loss of muscle mass and strength in older age is termed sarcopenia [2], which is associated with major adverse consequences including falls, dependency and the need for care, hospital admission, prolonged hospital stay and earlier death [3]. As muscle mass and strength are governed by environmental, genetic and epigenetic factors, the combination of these will determine whether an individual becomes sarcopenic, and how rapidly the condition will deteriorate. The genetic factors contributing to sarcopenia are likely to include those that contribute to muscle mass and strength in adulthood and identifying such factors and pathways will aid the identification of therapeutic approaches to prevent, mitigate or help reverse sarcopenia.

The renin-angiotensin system has gained significant attention as a potential target pathway to treat sarcopenia [4–8]. Angiotensin II (AII) promotes the skeletal muscle catabolic pathway (e.g., MuRF1) *in vitro* [9] and inhibits IGF-1 production [10]. Infusion of angiotensin into mice promotes skeletal atrophy [11]. In one small before and after clinical study of patients with heart failure, 6 months treatment with ACEi or AII antagonists increased exercise performance [12]. In cross sectional studies of individuals on antihypertensive therapies, ACEi usage associated with preservation of muscle mass and strength [13, 14]. Other studies have combined ACEi with exercise in both individuals with COPD and women with sarcopenia to attempt to identify combinatorial effects but the results of these trials do not provide a consistent picture of increased effectiveness of exercise interventions in those receiving ACEi [15–17].

These data suggest that reducing ACE activity and thereby AII could preserve muscle mass and strength in older individuals and one interventional study has supported this suggestion [18]. However, other trials in patients with COPD [4] and in patients with hypertension [19] have not shown a beneficial effect of ACEi. We recently conducted and published a multicentre 2 x 2 factorial RCT of leucine and ACE inhibitors in older people with sarcopenia (the LACE trial) which also showed no overall benefit from perindopril (an ACEi) therapy [20]. Whilst recruitment to this trial was low (as discussed in [21]) resulting in early termination by the funder, a meta-analysis of similar trials also showed a lack of effect, supporting the findings [20].

Studies of genetic variation show that the relationship between ACE activity and strength is not as simple as lower ACE activity associating with increased strength. One well-studied ACE promoter polymorphism, the insertion (I)/deletion (D) polymorphism, affects ACE synthesis with the D allele associated with greater ACE synthesis and higher circulating ACE activity [22]. However, contrary to the effects of AII on muscle mass and strength, several studies associate the ACE D allele with greater muscle mass and strength. For example, the D allele has been associated with increased performance in power sports [23], with increased strength following exercise training [24], and with increased strength in COPD patients [25]. Conversely, the I allele is associated with endurance [26, 27]. However, not all studies show the same enrichment of the D allele with strength in elite sports [28]. The effects of this polymorphism on muscle performance in the older population is unclear. In a study of 65-year-old Koreans the II genotype was associated with increased fatigue resistance [29]. In an Indonesian population of over 60 years the DD genotype was associated with reduced muscle mass [30] and in a Spanish cohort, individuals carrying the I allele had greater hand-grip strength than those with the DD genotype [31]. Conversely, in a large study of older Americans, (70–79 years) who continued to exercise, individuals with the II phenotype were more likely to show a loss of functional capacity [32]. One recent study of older individuals failed to identify any differences in lower limb strength normalised for body weight [33] and one further study failed to see any effect of the ACE I/D polymorphism on the exercise response in women over 60 [34].

These effects of the I/D polymorphism could be explained by variations in muscle fibre proportions. Skeletal muscle can be broadly categorised into two major fibre types (oxidative type I and more glycolytic type II). Type I fibres are used in endurance activities, whereas type II fibres are associated with power activities. In untrained volunteers, the D allele is associated with a greater proportion of type II fibres, with the I allele associated with a greater proportion of type I fibres [35], and treatment of patients with ACEi for 6 months increases MHC I (Myh7) expression [12]. Thus, over extended periods of time, higher ACE activity within the normal range, appears to promote a higher type II fibre proportion and so higher strength. However, more recent data have suggested that in Japanese individuals the opposite association between ACE genotype and muscle fibre type exists [36].

Type II fibres are more sensitive to both anabolic and catabolic stimuli than type I fibres [37, 38]. This higher sensitivity may contribute to the general loss of type II fibres that occurs in older individuals. However, this shift towards type I fibres is complicated by a shift in the opposite direction (increased proportion of type II fibres) that accompanies reduced physical activity and is seen in many patients with chronic disease [39].

It is therefore difficult to predict the effect of ACE genotype on the likelihood of sarcopenia as DD individuals may have started with a larger muscle mass and greater strength with a higher type II fibre composition that is more susceptible to loss. The effects of these polymorphisms may have contributed to the findings of the LACE study by affecting the muscle mass of individuals before the study or by modifying the response to therapy. In this study therefore, we analysed the genotypes of individuals with sarcopenia, enrolled into the LACE study to determine any association of ACE genotype with strength or muscle mass. We also determined the effect of genotype on the change in muscle mass, strength and physical performance 1 year later in those who completed the trial.

## Materials and methods

### Participants and physiological analysis

Individuals over the age of 70 with sarcopenia according to the EWGSOP definition (2010) [40] were recruited at 14 UK centres between April 2016 and December 2019 to a 2x2 factorial trial of leucine and/or ACE inhibition (trial registration ISRCTN90094835). Full recruitment criteria and methodology are described in [20]. The trial was performed in accordance with the ethical standards laid down in the 1964 Declaration of Helsinki and its later amendments and approved by the East of Scotland NHS research ethics committee (approval 14/ES/1099) and the UK Medicines and Healthcare Regulatory Authority (EudraCT number 2014-003455-61; Clinical Trial Authorisation number 36888/0001/001-0001). The study was a double-blind randomised controlled trial and sample preparation and analysis was carried out without any access to information that could identify the individuals involved. The trial protocol and primary outcomes have been published [20, 41]. The CONSORT diagram for the study with details of recruitment and dropout from the trial is published [20, 41]. Participants invited to attend for a screening visit had their age, weight and height measured and bioimpedance was used to measure overall muscle mass as part of the recruitment process. Bioimpedance was used at this stage to reduce the total number of DXA scans required. Individuals conforming to the EWGSOP guidelines as sarcopenic were recruited and attended a baseline visit. At this visit, their appendicular body composition (muscle mass, fat mass and bone mass) was determined by DXA and their physical performance measured. Physical performance measures were 6-minute walk distance (6MW) and short physical performance battery (SPPB), grip strength (measured using a hand-held Jamar dynamometer) and leg strength (Quadriceps Maximum Voluntary Contraction QMVC, measured as isometric voluntary knee extension with a Lafayette 01165 dynamometer Lafayette Instrument, Lafayette, IN, USA). Measurements were taken with the participant seated, the knee joint at 90 degrees, and with a non-elastic strap running between the chair and the ankle to restrain the dynamometer. The patients were randomised to placebo, leucine, perindopril or both leucine and perindopril at this baseline visit. Appendicular body composition and all physical performance measures were repeated 12 months later for those completing the trial. The LACE trial did not attempt to calibrate DXA readings between different sites or machines and this may have increased the noise inherent in the DXA results. However, each participant had their baseline and 12-month DXA scans performed on the same machine, removing this as a source of error from the longitudinal data. QMVC was divided by leg muscle mass to give a normalised quadriceps strength (kg/kg). Proportionate change was calculated as 12-month value/baseline value for each variable with longitudinal data.

The original study was powered to show difference in SPPB, but the study was terminated early due to poor recruitment. Of the 145 individuals recruited to the study 15 dropped out prior to a 3 month visit and their baseline samples were not retained for this study. All available samples were included in this analysis.

### DNA analysis

Whole blood samples were taken at the first study visit and frozen. DNA was extracted using the QIAamp DNA blood mini kit according to the manufacturer’s instructions. DNA genotyping was performed by PCR using the primers ACE I/D-F CTGGAGACCACTCCCATCCTTTCT and ACE I/D-R GATGTGGCCATCACATTCGTCAGAT as described [42]. The reaction mix was made using HOTstarTaq DNA polymerase (Qiagen) included 2pmol of each primer. The samples were amplified for 40 cycles using a cycle of 95C for 1 min 58 C for 1 min and 72 C for 1 min in a VertiPro 96 well thermocycler. The PCR products were separated by agarose gel electrophoresis through a 1.5% agarose gel, stained with SYBRsafe and visualised on a GelDoc imager (Biorad). All available samples from the study were included.

### Statistical analysis

The original study was a double-blind randomised controlled trial and sample preparation and analysis for this work was carried out without any access to information that could identify the individuals involved. Statistical analysis was performed in Aabel 3.0 and SPSS with the exception of the Chi squared test. Normality was determined by Shapiro-Wilk test, with differences between groups determined by Mann-Whitney U test for data that was not normal distributed and Student’s T-test for normally distributed data. Changes in strength at baseline and follow-up were determined by Wilcoxon matched-pairs signed rank test in Aabel 3.0. Values are given as median (interquartile range) for non-parametric data or as mean ± SD for normally distributed data. The threshold for statistical significance (alpha) was taken as 0.05. All available data was used for each comparison and no manipulations were performed to include missing values for individual tests. As the analysis here is exploratory, no corrections have been made for multiple testing. Reasons for loss to follow-up is detailed in [20, 41].

## Results

All individuals enrolled into the LACE study were sarcopenic as defined by EWGSOP and the demographics of the cohort are given in Table 1. Therefore, to establish an allele frequency for the ACE genotype in the UK population, the genotypes of controls from 13 published studies with a UK only population were averaged [43–55]. The ages of individuals and the study from which they are taken, and the details of the cohort used are given in S1 Table. This identified a total of 9909 genotypes (Table 2) and showed that the relative frequency of the alleles was I = 0.473 and D = 0.527. In the entire LACE cohort there was a significant over representation of the D allele (LACE population I = 0.411, D = 0.588, Chi^2^ = 3.97, p = 0.046). This difference was due to an excess of the D allele in the men but not the women (LACE men; I = 0.353, D = 0.647, Chi^2^ = 6.70, p = 0.010, LACE women; I = 0.458, D = 0.542, Chi^2^ = 0.14, p = 0.708, Table 2). Restricting the UK population to just men using the studies where sex and genotype were identifiable, gave a genotype frequency of I = 0.477, D = 0.523. However, this was from a smaller number of individuals (n = 710, II = 157, ID = 364, DD = 189) and the LACE male distribution was even further from the allele frequencies in this group than from the whole UK cohort (Chi^2^ = 7.10, p = 0.008). Restricting the UK cohort to those studies of individuals with an average age over 75 (II = 144, ID = 308, DD = 204) gave allele frequencies of I = 0.454, D = 0546. The men in the LACE cohort were still significantly different to this cohort (Chi^2^ = 4.85, p = 0.028).

**Table 1:** Demographics of the cohort.

	Women	Men
**number**	72 [52]	58 [47]
**Age (years)**	78 (75, 82)	77 (74, 84)
**weight (kg)**	63.0 (56.9, 71.0)	80.2 (73.5, 90.1)
**height (cm)**	157 ± 6	171 ± 7
**BMI (kg/m^2^)**	26.1 (23.2, 28.2)	27.2 (25.4, 30.5)
**SARC-F**	3 (4, 5)	4 (3, 4)
muscle mass (kg/m^2^)	5.74 (5.44, 5.99)	7.23 ± 0.64
**upper limb fat mass (kg)**	3.14 (2.45, 3.78)	2.64 (2.23, 3.17)
**upper limb muscle mass (kg)**	3.38 ± 0.56	5.92 ± 0.96
**lower limb fat mass (kg)**	9.24 (7.52, 11.88)	6.48 (5.41, 8.72)
**lower limb muscle mass (kg)**	10.66 ± 1.63	15.87 ± 2.46
**SPPB baseline**	7.0 (5.8, 9.0)	7.5 (5.0, 9.0)
**SPPB 12 months**	8.0 (5.0, 9.3)	8.0 (6.0, 9.0)
**Grip strength baseline (kg)**	13.6± 3.6	22.4 ± 5.4
**Grip strength 12 months (kg)**	15.0 ± 3.3	23.4 ± 5.9
**QMVC baseline (kg)**	9.9 (6.7, 13.0)	15.7 (11.9, 21.1)
**QMVC 12 months (kg)**	10.4 (7.6, 13.4)	15.0 (12.0, 18.5)

Numbers given in square brackets are the number of individuals completing the trial. BMI: body mass index, SARC-F: Strength, assistance with walking, rising from a chair, climbing stairs, and falls questionnaire score, SPPB: Short Physical Performance Battery score: QMVC: Quadriceps Maximal Voluntary contraction.

**Table 2:** Genotypes of the cohort.

	Genotype						
Group	II	ID	DD	I	D	Chi^2^	P Value
**Controls**	2200	4967	2742	0.473	0.527		
**LACE**	21 (29)	65 (65)	44 (36)	0.411	0.588	3.97	0.046
**Women**	15 (16)	36 (36)	21 (20)	0.458	0.542	0.14	0.708
**Men**	6 (13)	29 (29)	23 (16)	0.353	0.647	6.70	0.010

Predicted numbers using the control allele frequencies are given in brackets

Chi^2^ was calculated on allele number (degrees of freedom = 1)

**Fig 1:**
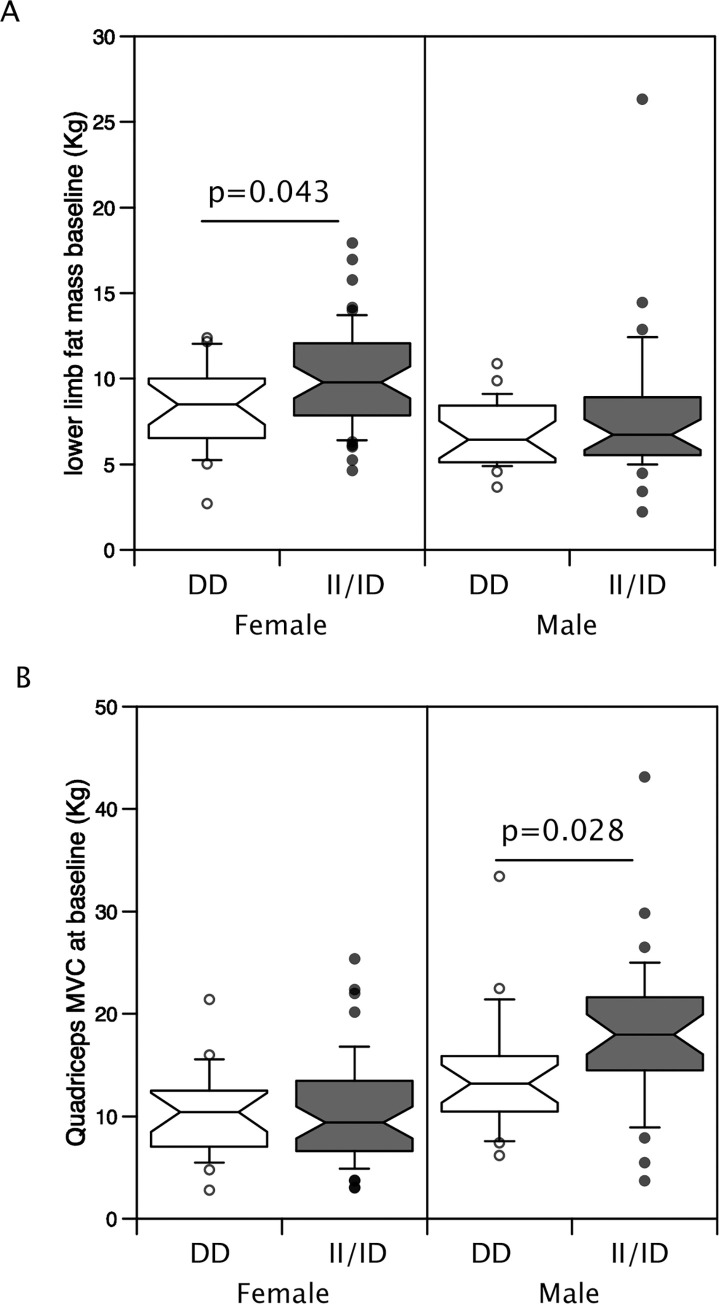
Effect of ACE genotype on lower limb fat and muscle strength. Limb composition was determined by DXA in 72 women and 58 men. The individuals were genotyped for the ACE I/D polymorphism and the effect of polymorphic variation on body composition and strength was determined. The II/ID genotypes were associated with a higher lower limb fat mass than the DD genotype in women but not in men (A). In men but not women, the II/ID genotypes were associated with greater quadriceps muscle strength than the DD genotype. (p Values calculated by Mann-Whitney U test).

Given the difference in body composition and strength between men and women, the sexes were analysed separately. Furthermore, due to the small number of II homozygous individuals, the cohort was analysed as those possessing the minor (I) allele and DD individuals. In women only, the I allele was associated with higher fat mass in the lower limbs but not with upper or lower limb muscle mass or strength (Table 3, Fig 1). This difference in fat mass was not seen at 12 months in the individuals who completed the trial.

**Table 3:** Baseline body mass composition and muscle physiology in the different genotypes split by sex.

	Women			Men		
	DD	ID/II	P value	DD	ID/II	P value
**n**	21	51		23	35	
**Weight (kg)**	62.87 ± 12.51	64.45 ± 10.56	0.780	82.00 (73.70, 90.05)	80.20 (73.55, 88.50)	0.937
BMI (kg/m^2^)	25.06 (21.87, 27.04)	26.16 (24.09, 28.60)	0.232	28.41 (26.09, 30.11)	27.11 (25.27, 30.40)	0.567
**Height (m)**	157 (154, 163)	157 (152, 161)	0.289	169 (167, 174)	173 (167, 177)	0.373
muscle mass (kg/m^2^)	5.59 (5.29, 6.00)	5.79 (5.53, 5.98)	0.295	7.43 (6.79, 7.62)	7.17 (6.82, 7.79)	0.855
**upper limb fat mass (kg)**	2.73 (2.23, 3.51)	3.21 (2.26, 3.83)	0.511	2.52 (2.03, 3.02)	2.78 (2.28, 3.25)	0.131
**lower limb fat mass (kg)**	8.51 (6.54, 10.02)	9.79 (7.85, 12.07)	0.043	6.45 (5.20, 8.11)	6.74 (5.54, 8.89)	0.666
**upper limb muscle mass (kg)**	3.41 (3.09, 3.79)	3.35 (2.92, 3.80)	0.511	5.66 (5.09, 6.49)	5.96 (5.15, 6.53)	0.435
**lower limb muscle mass (kg)**	10.76 ± 1.22	10.62 ± 1.78	0.712	16.00 ± 1.63	15.79 ± 2.91	0.749
**SPPB baseline**	7.0 (6.0, 9.0)	7.0 (5.0, 9.0)	0.975	8.0 (6.0, 9.0)	7.0 (5.0, 9.0)	0.315
**Grip strength baseline (kg)**	13.9 ± 3.6	13.5 ± 3.6	0.593	22.8 ± 4.1	22.1 ± 6.1	0.595
**QMVC baseline (kg)**	10.4 (7.1, 12.4)	9.4 (6.6, 13.5)	0.796	13.2 (10.5, 15.9)	18.0 (14.5, 21.6)	0.028
**QMVC/leg muscle mass (kg/kg)**	0.93 (0.67, 1.19	0.93 (0.65, 1.19)	1.000	0.78 (0.63, 1.15)	1.18 (0.86,1.33)	0.035

BMI: body mass index, SPPB: Short Physical Performance Battery score: QMVC: Quadriceps Maximal Voluntary contraction. Normally distributed data are presented as mean ± SD with significance of differences determined by t-test. Non-parametric data are presented as median (inter quartile range) and the significance of differences was determined by Mann Whitney U test

**Fig 2:**
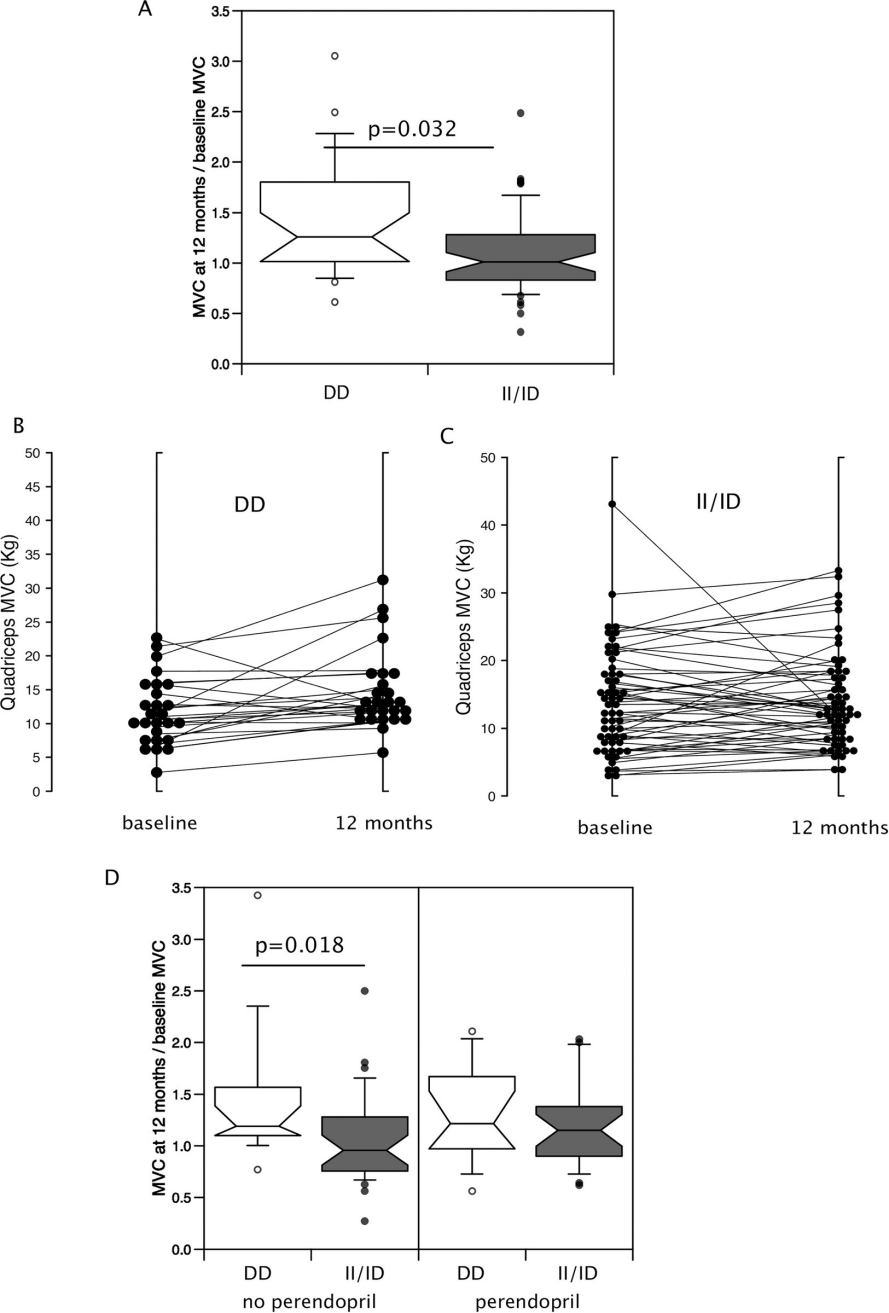
Effect of genotype on change in strength over 12 months of the LACE trial. Proportionate strength was determined by dividing MVC at month 12 by MVC at baseline allowing us to combine data from men and women in the study. (A) Individuals with the DD genotype had a higher proportionate strength at month 12 than individuals with the II/ID genotype (Mann-Whitney U test). (B and C) Comparison of the baseline and 12-month MVC showed that the DD individuals (B) increased in strength over the course of the trial (p = 0.003 Wilcoxon-signed rank test) whereas there was no change in strength in the II/ID individuals (C). There was no effect of perindopril on proportionate strength in either the DD or II/ID genotypes (D).

In men, presence of the minor allele was associated with higher QMVC both as a raw value (II/ID median 18.0 kg IQR (14.5, 21.6) vs DD 13.2 kg (10.5, 15.9) p = 0.028 Fig 1, Table 3) and when normalised to leg muscle mass determined by DXA (II/ID 1.18 kg/kg (0.86,1.33) vs DD 0.78 kg/kg (0.63, 1.15) Table 3, p = 0.035). However, there was no difference in lower limb muscle mass between the genotypes. Using proportionate change in muscle strength over 12 months to allow the combination of both men and women showed that the DD genotype was associated with a greater average increase in muscle strength over the 12-month period (% change in QMVC: II/ID median = 3.8% (-21.1%- 30.6%), DD median = 20.8% (8.8%-66%), p = 0.032, Fig 2A). To confirm this analysis comparing QMVC at baseline and follow-up using a Wilcoxon matched-pairs signed rank test within the genotypes showed that the DD individuals increased in strength (baseline 10.8 kg (7.6, 15.8), vs 12 months 12.8 kg (10.5, 17.4), p = 0.003) over the period of the trial whereas the II/ID individuals did not (baseline 13.0 kg (8.1, 18.1), vs 12 months 12.2 kg (8.6, 17.8), p = 0.504, Fig 2B and 2C). However, there was no accompanying change in leg muscle mass (DD individuals; baseline 14.6 kg (11.5, 15.9) vs 12 months 14.3 kg (11.3, 15.4) p = 0.322, II/ID individuals; baseline 12.1 kg (10.4, 15.5) vs 12 months 12.5 kg (10.5, 15.9) p = 0.808).

To determine if the increase in muscle strength was a consequence of the treatment with perindopril, we compared QMVC at 0 and 12 months in II/ID and DD individuals who completed the study. This analysis showed that, consistent with the overall trial data, perindopril did not cause the increase in strength in the DD genotype individuals or the II/ID individuals as specified groups. Wilcoxon matched-pairs analysis showed that the DD individuals on perindopril did not increase their strength over 12 months (change in QMVC = 2.55 kg (-0.35, 4.15) p = 0.196) but those not on perindopril did (change in QMVC = 1.8 kg (1.2, 6.6), p = 0.008). However, strength did not increase in II/ID individuals whether on perindopril (change in QMVC = 1.45 kg (-2.3, 3.9), p = 0.247), or not (change in QMVC = -0.4 kg (-2.6, 3.3), p = 0.860). In the absence of perindopril, the increase in proportionate strength was larger in individuals with the DD than the II/ID genotype but there was no difference in the presence of perindopril (Fig 2, Table 4). No differences were seen in the change in QMVC between baseline and follow-up in either DD or II/ID genotypes in response to leucine.

**Table 4:** Effect of perindopril on muscle performance based on genotype.

	DD			ID/II		
	perindopril	No perindopril	P value	perindopril	No perindopril	P value
**n**	15	17		30	37	
**f/m**	5/10	8/9		17/13	22/15	
**SPPB baseline**	8 (6, 9)	7 (6, 8)	0.635	8 (5, 9)	7 (5, 9)	0.896
**SPPB 12 months**	9 (5, 10)	9 (6, 9)	0.710	8 (4, 9)	8 (6, 9)	0.246
**Change in SPPB**	0 (-1, 2)	1 (-1, 2)	0.602	0 (-2, 1)	1 (0, 2)	0.017
**Proportionate upper limb muscle mass at 12 months**	1.01 (0.94, 1.05)	1.01 (0.97, 1.05)	0.821	1.03 (0.98, 1.07)	1.00 (0.96, 1.04)	0.303
**Proportionate lower limb muscle mass at 12 months**	1.00 (0.99, 1.03)	1.01 (0.99, 1.05)	0.545	1.01 (0.95, 1.05)	0.99 (0.96, 1.02)	0.692
**Proportionate grip strength at 12 months**	0.97 (0.90, 1.19)	1.04 (0.92, 1.32)	0.423	1.07 ± 0.33	1.01 (0.93, 1.11)	0.898
**Proportionate QMVC at 12 months**	1.21 (0.97, 1.67)	1.19 (1.10,1.57)	0.667	1.15 (0.90, 1.38)	0.96 (0.76, 1.27)	0.217

SPPB: Short Physical Performance Battery score: QMVC: Quadriceps Maximal Voluntary Contraction. Data are presented as median (inter quartile range) and the significance of differences was determined by Mann Whitney U test

Finally, we compared the effect of perindopril on physical performance measured by the short physical performance battery (SPPB). In those with the II/ID genotype there was a median increase in SPPB of 1 point over the 12 months in the absence of perindopril but in the presence of perindopril median change in SPPB was 0 points (Table 4). In the DD genotype group, there was no change in SPPB at 12 months. Leucine had no effect on SPPB in either DD or II/ID genotypes.

## Discussion

Our data show that, in the LACE cohort of sarcopenic individuals, the number of men carrying the I allele (II and ID combined) was lower than predicted from the general UK population. The I allele frequency in men in our study is 0.353 compared to the expected 0.474. Furthermore, we found that II/ID men had greater muscle strength than those with the DD genotype. This observation is opposite to the effects of the I/D polymorphism seen in the non-sarcopenic population where the D allele is found enriched in power athletes and is associated with greater muscle mass and strength in a number of diverse cohorts including those representing elite athletes, normal healthy individuals and patients with chronic obstructive pulmonary disease [23–25]. The reduction in the number of men carrying the I allele could be explained by one of three main mechanisms, survivor bias, recruitment bias or a resistance to sarcopenia for individuals with at least one I allele. A survivor or recruitment bias due to II males having died or been too frail to participate is unlikely given the body of literature indicating the association of the D allele with both morbidity and mortality [56–62]. However, although comparison with published UK studies where the individuals had average age over 75 did not affect our results, a recent meta-analysis of studies including centenarians, and older individuals as well as younger controls suggests a survivor advantage for DD individuals but not for ID individuals [63]. For this bias to account for our findings on the disproportionate number of DD men, the survivor effect would need to be greater and/or observed earlier in men as our observation of increased DD individuals was restricted to men. Given the size of our study this finding needs to be replicated in a larger study. It is also possible that recruitment bias occurred as a consequence of differences in the effects of EWGSOP algorithm in men and women. Rather, our finding would be consistent with II males being relatively resistant to sarcopenia, and therefore under-recruited. This possibility is consistent with the original hypothesis that lowering ACE activity would associate with increased muscle strength but if this was the major mechanism, it might be expected that further lowering ACE activity would have increased muscle strength and this did not happen. An alternative mechanism that could contribute to this resistance would be the higher type I fibre proportion associated with the I allele compared to D allele and the relative insensitivity of type I fibres to atrophy compared to type II fibres [35]. Therefore, individuals with the II genotype could start with a lower muscle mass and strength than their DD counterparts but be more likely to maintain muscle mass and strength and so less likely to become sarcopenic. The ability to maintain physical performance/endurance because of the greater proportion of type I fibres may also contribute to greater relative physical activity over time helping to maintain muscle mass. A reduced rate of muscle loss would also account for the reduced strength seen in the sarcopenic DD men compared to those carrying the I allele. Again, this reduced strength in DD men is counter to the situation in non-sarcopenic populations that have been studied which show higher strength in those with the DD genotype.

We did not observe either of these effects in women. Previous studies have shown that women have a higher proportion of type I fibres in the quadriceps compared to men [64, 65] suggesting that the effects of the II genotype may make a smaller difference to the overall proportion of fibres in women than in men. It is also possible that the effects of AII are enhanced by testosterone. For example, testosterone has been shown to contribute to the AII dependent increase in blood pressure in male rats [66], but any such cross talk in skeletal muscle remains to be established.

The LACE trial, from which these samples were taken, did not report any effect of perindopril on muscle mass or strength and a meta-analysis performed following the trial also concluded that ACE inhibition did not increase muscle mass or strength. In this analysis we show that there is no positive effect of ACE inhibition either in individuals with the II/ID genotypes or the DD genotypes although we did see an increase in muscle strength in DD individuals enrolled in the trial, whether or not they were on perindopril or leucine. The presence of this increase in strength may have been a consequence of a combination of the two agents that would have been identifiable had the trial been allowed to recruit its full cohort, a consequence of altered activity as a response to being recruited to a trial or potentially a type II error given the size of the trial. The reason that it is only observed in the DD group could suggest either that this group is most likely to respond to both activity and potentially to drug therapies due to a higher proportion of type II fibres or that as these individuals started with a lower QMVC there was a greater response to any increase in activity. Physical activity was not measured in this study. Whether trial recruitment acted as a trigger to increase activity would not have been detectable as it would not be possible to quantify pre-recruitment activity.

Consistent with a change in behaviour, individuals carrying the I allele showed an increase in SPPB by 1 point over the 12 months. Both changes (increased strength in DD individuals and increased SPPB in II/ID individuals) only occurred in the absence of perindopril. It is not clear why this change was restricted to those not on perindopril but given the drop-out rate was higher for those on perindopril than those not taking perindopril, it is possible that adverse responses to perindopril treatment reduced the effect of being recruited to a trial.

The LACE trial and a meta-analysis of available randomised controlled trials did not identify any effect of ACEi on muscle mass or strength (33), one possible reason for this is that the trial did not combine pharmacological treatment with exercise. In support of this possibility some studies have suggested that exercise responses are larger in patients taking ACEi than in those not taking ACEi [67] and some but not all animal studies have shown a that ACEi increases the gains from exercise [68, 69]. However, several randomised control trials of exercise have not found support for this proposal. Indeed, in patients with COPD combining ACEi with exercise reduced the efficacy of the exercise program (measured as peak work rate [16]) and in healthy volunteers, ACEi impaired lean muscle mass gain, reduced left atrial volume and reduced haemoglobin content [70]. Furthermore, in older individuals with functional impairment there was also no increased positive effect of ACEi with exercise [5]. The effects of genotype were not addressed in these studies, but the findings from our study would be most consistent with ACEi reducing any effect as the only improvements we saw (increase in SPPB in II/ID individuals and increase in strength in DD individuals) were in the group not taking perindopril.

### Limitations of this study

This study is limited due to its very small sample size and therefore requires repeating in a larger study. The trial also only recruited individuals with sarcopenia making us reliant on allele frequencies from previous studies rather than a control cohort recruited at the same time and from the same communities. To overcome this latter problem, we identified studies with control cohorts of UK individuals to calculate an allele frequency that was representative of the general UK population. Furthermore, as the group sizes are small and the study was a 2x2 factorial trial it is possible that there was an effect of perindopril on strength in the II/ID group, but this is too small to detect in this study and is complicated either by the treatment with leucine or that inclusion in the trial leads to altered physical activity.

In conclusion our analysis suggests that sarcopenic men with the DD ACE genotype may be weaker than men with at least one I allele. We speculate that this is due to differences in the fibre types that have been shown to occur with the differences in genotype and the higher sensitivity of type II fibres to atrophy than type I fibres. The data imply that genotype does not account for the lack of effect of perindopril that we observed in the LACE trial. However, if the basis of the effects of ACE genotype is due to long-term effects of lower ACE levels on fibre type, it is possible that one year of treatment is not sufficient to alter the fibre proportions sufficiently to reduce the loss of strength. It is also possible that the number of type II fibres has been reduced to such an extent that fibre shift is no longer a viable route to increase muscle function.

## Supporting information

S1 Checklist STROBE statement—Checklist of items that should be included in reports of observational studies. (DOCX)

S1 Table (XLSX)
